# Observational Study on Lameness Recovery in 10 Dogs Affected by Iliopsoas Injury and Submitted to a Physiotherapeutic Approach

**DOI:** 10.3390/ani11020419

**Published:** 2021-02-06

**Authors:** Giuseppe Spinella, Benedetta Davoli, Vincenzo Musella, Ludovica Dragone

**Affiliations:** 1 Department of Veterinary Medical Sciences, University of Bologna, Ozzano dell’Emilia, 40064 Bologna, Italy; benedettadavoli@gmail.com; 2 Department of Health Sciences, University of Catanzaro, 88100 Catanzaro, Italy; musella@unicz.it; 3 Physiotherapy and Rehabilitation Center “Dog Fitness”, Via Fabio Filzi, 32, 42124 Reggio Emilia, Italy; ludovica@dogfitness.it

**Keywords:** iliopsoas muscle, rehabilitation, muscle injury, border collies, dog

## Abstract

**Simple Summary:**

Acute trauma of the iliopsoas muscle is often observed in sports and very active dogs. Nevertheless, the diagnosis is not always correctly addressed and can be underestimated during a clinical visit because it is generally secondary to other orthopedic and neurological issues. However, a correct diagnosis can lead to complete recovery of lameness in dogs who submit to physiotherapy. This paper has investigated, in a preliminary observational study, the recovery time for dogs affected by primary iliopsoas lesions and their return to normal activity.

**Abstract:**

This preliminary study has investigated the outcome of physiotherapy in 10 dogs affected by a primary lesion of the iliopsoas muscle and the potential effects of sex, age, and breed on recovery. Ten dogs with primary injury of the iliopsoas muscle were retrospectively included in this study. Dogs were submitted to a rehabilitation program, characterized by a multimodal approach, including physical therapies and modalities. After recovery, patients were submitted to a further support period of rehabilitation to promote muscle strengthening and limit injury recurrence during their return to normal functional and sports activity. Border collies were highly represented. The recovery of lameness occurred after a mean of 22.6 ± 14.7 (median 18) days with a median number of five sessions. None of the examined variables affected the recovery time, except for the border collie breed, which showed a significantly faster recovery time; however, there was no difference between the breeds with regard to the number of sessions. Multimodal rehabilitation therapy may promote lameness recovery of mild-to-moderate iliopsoas lesions within 3 weeks. This preliminary study reports a clinical approach and recovery of primary iliopsoas lesions, findings that can provide clinicians with useful prognostic information for dogs involved in sports activities.

## 1. Introduction

The iliopsoas muscle (*Musculus iliopsoas*) is composed of two different muscles: *Musculus psoas major* and *Musculus iliacus* [1,2]. Injuries of the iliopsoas muscle frequently occur in sports dogs (agility dogs, flyball dogs, disc dogs, etc.), and they can be acute or chronic, mainly secondary to trauma [3]. The history of dogs affected by iliopsoas muscle lesions generally includes the presence of hindlimb lameness of various grades. Lameness can appear intermittently after a physical activity, with limited weight-bearing. Moreover, dogs with mild injuries may experience a gradual improvement over time, but a decrease in performance during training or competition is often reported [3,4]. Lesions of the iliopsoas muscle are generally classified into three grades based on severity: grade 1 (mild), grade 2 (moderate), and grade 3 (severe) [5,6,7].

Orthopedic examination reveals pain during hindlimb extension and simultaneous internal or external rotation of the hip joint or during manual pressure on the tendon insertion at the level of the lesser trochanter [8,9]. The induced pain is generally very evident, abrupt, and severe [10]. Moreover, impairment of the femoral nerve can be observed with symptoms of neuropathy associated with a reduction of the patellar reflex and ipsilateral proprioceptive sensitivity to the lesion, weakness of the posterior train, and atrophy of the quadriceps femoral muscle [11]. Diagnostic imaging is also used; it can show the degree and the site of the muscle lesion. These techniques mainly include ultrasound and magnetic resonance imaging, frequently associated with laboratory tests, although these tests are often non-specific for particular lesions [6,12].

Physiotherapy with a multimodal approach (appropriate rest, manual therapies, the application of various modalities, and owner education) can positively influence the healing of muscle injuries and abrogate the clinical signs [8]. However, there is a lack of specific publications reporting the recovery times in active dogs affected by iliopsoas injury submitted to rehabilitation. This information should provide clinicians with crucial prognostic information for owners and trainers.

The aim of this preliminary study was to evaluate the outcome of physiotherapy in dogs affected by primary lesions (with no other concomitant diseases) of the iliopsoas muscles to investigate the recovery times, in terms of days, and the required number of sessions from the start of rehabilitation. In particular, we have purposely interpreted “recovery” as the return to the previous lifestyle (normal activity for both athletes and pets) in the absence of symptoms (lameness and pain). We also compared this outcome with some specific variables, namely, age, sex, and breed.

## 2. Materials and Methods

We reviewed the medical records of dogs affected by primary injury of the iliopsoas muscles and referred to the physiotherapy and rehabilitation center “Dog Fitness”. Inclusion criteria were (a) dogs ≥ 12 months; (b) accurate physiatric examination, conducted by a licensed specialized veterinary practitioner in physiatric medicine with >5 years of experience; (c) positive painful response to diagnostic palpation of the iliopsoas and/or to hindlimb extension with internal or external rotation of the hip joint; (d) complete rehabilitation program until signs of recovery (absence of lameness and pain on palpation); and (e) no other concomitant orthopedic or neurological pathologies, correctly excluded by specialists.

For all patients, we recorded the breed, sex, age, sports activity (if athletic), date of the physiatric examination, degree of lameness at the first visit, number of days and sessions from the start of rehabilitation to lameness recovery and the total number of physiotherapy sessions, and days including the “maintenance”/”support” period (rehabilitation of physical support after recovery). We also recorded the body condition score (BCS) on a scale from 1 to 9.

During the physiatric examination, the patient’s algic response, both to direct palpation of the iliopsoas muscle and to hindlimb extension with internal or external rotation of the hip joint, were evaluated and considered clinically specific for the clinical diagnosis of iliopsoas injury. On dynamic examination, lameness was assigned a specific grade from 0 to 4 according to severity (0 = no lameness observed; 1 = mild: weight-bearing lameness; 2 = moderate: weight-bearing lameness with intermittent non-weight bearing; 3 = severe: non-weight-bearing lameness with brief intermittent weight-bearing; 4 = non-weight-bearing lameness at all times). An ultrasound examination to evaluate the extent of the injury was recommended to all owners.

The physiotherapy regimen for all dogs consisted of a minimum of two sessions per week and restricted physical activity; specifically, no running or jumping and only walking on a leash was suggested.

The physiotherapy approach comprised diathermy with a resistive capacitive energy transfer therapy (TECAR therapy, DVET900, White Medical and Beauty, Pieve di Cento (BO), Italy) at 20% power (1 MHz frequency and 750 W maximum power); therapeutic ultrasound (2Ultrasound Vet 1000, Globus Vet, Codognè (TV), Italy) at 1 MHz, 20% pulsed ultrasound and 0.6–0.8 W/cm^2^; passive range of motion (PROM) exercises; massage; and underwater treadmill (UWTM, Hydro Physio, Shropshire, UK). UWTM was included at an increasing intensity according to the patient’s response and at a minimum duration of between 5 and 10 min, with a variable speed depending on the size of the animal (up to a maximum of 2 km/h for a border collie) and the height of the water adjusted to the level of the greater femoral trochanter. The water temperature was maintained at about 33 °C to promote correct muscle work without excessive fatigue. The duration of UWTM treatment was then gradually increased, reaching a maximum of 20 consecutive minutes of work in the water. In some dogs, jets against the flow were introduced in the final UWTM rehabilitation sessions to promote muscle strengthening. Before each therapy session, the physiotherapist clinically evaluated the patient to record the patient’s health status and any improvement or complication.

Athletic dogs were gradually re-introduced to sporting activity according to the training protocol of the specific discipline. Introduction of adequate and longer pre-activity warm-up was highly recommended. Treatment was considered complete at the time of the disappearance of the lameness and pain on palpation.

When possible, a final ultrasound evaluation was performed before the animal returned to previous activities. However, a further rehabilitation support period was performed to avoid recurrence of the lesions. There was a gradual decrease in the treatments, with a longer period of time between each treatment.

Due to the retrospective nature of this clinical study, we did not perform any experimental protocols on animals (European directive 2010/63/UE, 22 September 2010). All procedures were approved by the owners and were performed during routine clinical examination carried out by certified licensed veterinarians following a normal standard protocol and guidelines accepted in veterinary clinical practice by the National Federation of Italian Veterinarians (FNOVI Deontological Guidelines, art. 15).

### Statistical Analysis

We submitted all data to descriptive statistical analysis to determine the mean, standard deviation (SD), and median for each variable. We used the Mann–Whitney U test to make comparisons between any specific variables (such as gender, age, and breed) in relation to the time to clinical recovery (absence of lameness) and number of therapy sessions. For age, we divided the sample into two groups—younger adults and older adults—based on the reported mean age. We set the significance level at *p* ≤ 0.05. We performed all statistical analyses using Stata version 15 (StataCorp, College Station, TX, USA).

## 3. Results

Based on the medical record analysis, we found 22 patients with iliopsoas myopathy disease, but only 10 met the inclusion criteria. The 10 patients were represented by four males and six females. The breeds were border collie (*n* = 7), mixed breed (*n* = 1), Belgian Malinois (*n* = 1), and Jack Russell terrier (*n* = 1). The mean age was 5.1 ± 2.79 (median 5) years, and the median BCS was 5 (ideal). Of the 10 included dogs, seven were athletic (two dogs practiced more than one activity): agility (*n* = 4), disc dogs (*n* = 3), sheepdog (*n* = 1), and obedience (*n* = 1). The remaining three patients did not practice any specific sport, but they had an active lifestyle (Table 1).

The average onset of lameness from trauma to diagnosis was 81 ± 95.10 days (median 30; the mean was 31.2 days if we exclude the two cases with longer periods of onset of 240 and 270 days); in two cases, the exact moment of the trauma was not known. The diagnosis of iliopsoas myopathy generally occurred during physiatric examination (*n* = 6), while in four cases it occurred 1 week (*n* = 2) or 2 months (*n* = 2) before the physiotherapy visit by an orthopedic specialist. However, all dogs were re-evaluated by a physiatrist before starting the rehabilitation program.

All dogs showed pain on palpation of the iliopsoas muscle, but only four exhibited pain on hindlimb extension with internal/external rotation of the hip joint. The pain on extension was resolved in all four dogs within 3-4 sessions. There was no limitation to hip ROM (50–160° for all dogs).

During the physiatric examination, grade 1/4 lameness was observed in eight dogs, one dog had grade 2/4 lameness, and one dog presented grade 4/4 lameness during work and grade 2/4 at walk.

Ultrasound examination was performed in five cases; this examination confirmed the clinical diagnosis of a moderate-grade lesion of the muscle–tendon junction.

The rehabilitation for all dogs began after the physiatric visit. The recovery of lameness occurred after 22.6 ± 14.7 (median 18) days with a median of 5 sessions. The median number of treatment sessions (including the maintenance period after lameness recovery) was 18, which was related to an average number of days from the physiatric visit to program completion of 130.6 ± 75.7 (median 91) days. No pharmacological therapies were administered during physiotherapy. No pain on palpation and hindlimb extension was observed at the time of lameness recovery.

**Figure 1:**
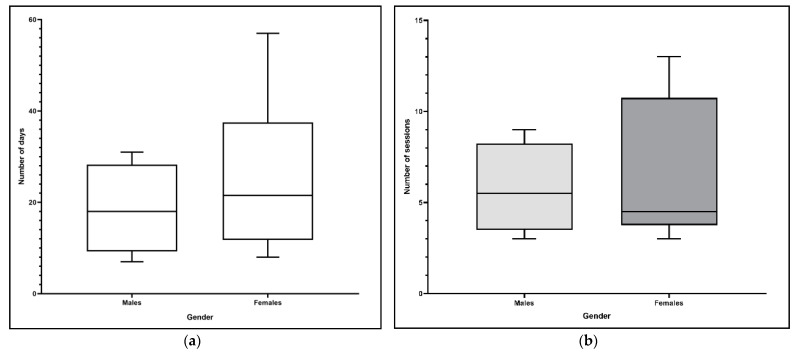
(**a**) Influence of gender on the number of days until lameness recovery (*p* = 0.81). (**b**) Influence of gender on the number of therapy sessions until lameness recovery (*p* = 0.99).

With regard to gender, there were no differences in response to the rehabilitation protocol in terms of the days and the number of therapy sessions (*p* = 0.81 and *p* = 0.99, respectively; Figure 1a,b).

**Figure 2:**
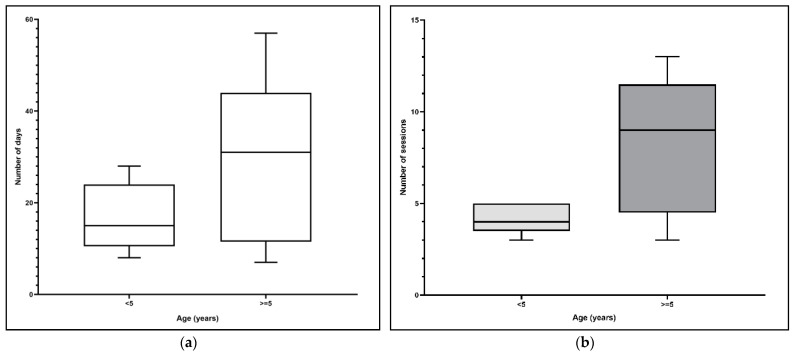
(**a**) Influence of age on the number of days until lameness recovery (*p* = 0.28). (**b**) Influence of age on number of therapy sessions until lameness recovery (*p* = 0.10).

To examine the effect of age, we used the mean age (5 years) to divide dogs into younger adults (<5 years) and older adults (>5 years). There were no differences between these groups with regard to the days and the number of therapy sessions (*p* = 0.28 and *p* = 0.10, respectively; Figure 2a,b).

**Figure 3:**
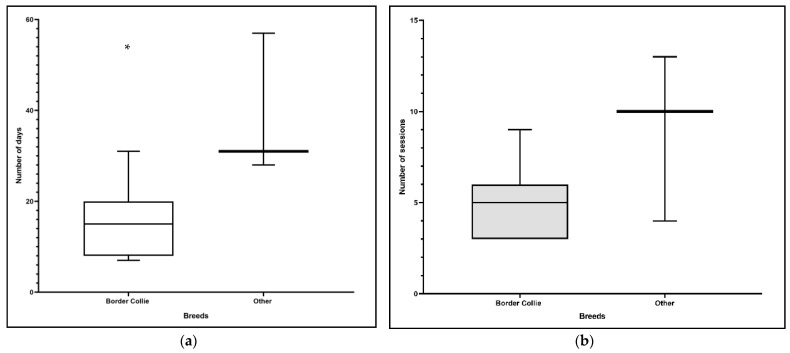
(**a**) Comparison of border collies and other breeds for number of days until lameness recovery (*p* = 0.04); * this is the only variable with a statistically significant difference. (**b**) Comparison of border collies and other breeds for number of therapy sessions until lameness recovery (*p* = 0.24).

Finally, we compared border collies with the other affected breeds and found a difference (*p* = 0.04) for the number of days from start of physiotherapy to recovery (Figure 3a). Specifically, border collies required fewer days for recovery. However, there was no difference between border collies and the other breeds for the number of therapy sessions (*p* = 0.24; Figure 3b).

## 4. Discussion

We investigated the recovery times for clinical signs in dogs affected by primary lesion of the iliopsoas muscles and submitted to physiotherapy. Furthermore, we observed whether specific variables (gender, age, and breed) could influence the healing period, measured in days, and the number of sessions from the start to the end of rehabilitation. Preliminary results showed that only breed (border collie) had a significant influence on the number of the days to recovery but not on the number of sessions. However, border collie was the most represented breed in this study; only three dogs were reported as “other breed”. This breed is widely involved in most canine sports [6,13], and it is the breed most commonly injured during agility, more than would be expected [3]. The border collie has always shown a great ability for training and cooperation with humans. It was first trained as a working dog because of its skill in performing long-lasting efforts with high-endurance resistance [14]. The fact that border collies recovered faster than the other breeds could be due to this breed’s positive attitude towards physical activity, which helps in the rehabilitation process because it allows the physiotherapist to immediately introduce an intensive approach. However, when we compared the average number of days between two single rehabilitation sessions, we found that the border collies were brought to the physiotherapy center after a shorter time interval (mean 3 days) compared with the other breeds (mean 4 days). This was not related to a difference in the therapeutic protocol program; rather, it was due to greater involvement of the owners, who were absolutely determined to obtain a faster positive outcome and a faster return to training.

The comparison related to sex (males and females) and age (younger and older dogs) variables did not show any statistically significant difference. Lack of difference between males and females was certainly predictable. In relation to age, we observed that, although this difference was not statistically significant, younger dogs showed a faster and more homogeneous recovery than the older ones. In the future, a larger sample could be useful to clarify the influence of age on recovery and in rehabilitation.

Furthermore, 70% of our cases were athletic dogs, and a traumatic injury had been reported in their history; this phenomenon has been reported in the literature [6,15]. Iliopsoas muscle strains are believed to occur during eccentric contraction, when the muscle is contracting while lengthening [4,16]. In particular, as reported by their owners, our patients showed a disinclination to jump and the onset of lameness following activity, with a decrease in sports performance. Previous studies have shown that the refusal to jump and low performance are recurrent and lead to early findings of iliopsoas injury in sports dogs [10,17].

Lameness associated with iliopsoas injury has been previously reported as mild, ranging between grade 1/4 and grade 2/4 [15]. The dogs included in this study presented a lameness grade similar to those reported in the literature. Only one case had an initial grade 4/4 lameness reported immediately after training. This dog was submitted to an ultrasound investigation, which showed a moderate/severe injury to the iliopsoas muscle. However, its early clinical status returned to that of the other cases (i.e., those with a mild-to-moderate injury) after five sessions of physiotherapy.

The clinical diagnosis of injury to the iliopsoas muscle is mainly associated with the pain response to manual compression of the iliopsoas tendon and to the hindlimb extension maneuver with internal and external rotation of the hip joint [10]. All dogs included in this study showed a positive response to these maneuvers. No limitation to hip ROM was observed, probably because of the absence of other concomitant pathologies.

Only five enrolled dogs underwent a muscle ultrasound due to financial limitations of the owners. This imaging technique allows diagnostic confirmation of the lesion as well as its characterization in terms of lesion position and width (muscle–tendon junction, muscle body, and possible involvement of the femoral nerve) [10]. All examined lesions were located mainly at the muscle–tendon junction of the iliopsoas muscle. This lesion is commonly characterized by a poor visualization of the muscle–tendon fibers, with a moderate-to-severe decrease in echogenicity [18]. Cullen and collaborators [6] reported similar findings in dogs with traumatic injury to the iliopsoas muscle. No other advanced diagnostic imaging exams were performed in our cases [6]. X-ray examination could be useful if a chronic lesion is suspected, because it may visualize mineralized areas and/or tendon avulsion with avulsion fracture. However, this diagnostic technique has a limited accuracy in evaluating acute lesions [19,20]. Advanced diagnostics such as computed tomography and magnetic resonance imaging may be used to diagnose iliopsoas strains [21,22,23]. Although computed tomography is valuable for imaging soft tissue lesions, the use of magnetic resonance imaging provides greater diagnostic accuracy for muscle lesions [23]. However, higher costs of instrumentation purchases and maintenance and essential general anesthesia for dog examinations could limit its application. Conversely, sedation is not always required for ultrasound examination [10,18].

Several conservative and surgical treatments to promote recovery from iliopsoas injury have been reported in the literature [6,24,25]. However, physiotherapy appears to be the most correct conservative approach to mild or moderate muscular injuries. In our study, manual and instrumental physiotherapy approaches were scheduled twice a week. Other studies have reported that in early stages it could be useful to perform up to three sessions per week [8]. However, the dogs included in our study had mild-to-moderate injuries. Sessions were gradually reduced to one per week in relation to the clinical status of different patients. Physiotherapeutic approaches may include massage, controlled PROM exercises, stretching exercises, laser therapy, electrical muscular stimulation, therapeutic ultrasound, diathermy, and UWTM [10,17]. During rehabilitation, all dogs in this study were submitted to massages and PROM exercises in the first part of the session and then to modalities including diathermy by TECAR therapy, therapeutic ultrasound, and UWTM, always performed in this specific order. During the rehabilitation phase, an initial period of physical activity restriction was also recommended. In this study, the protocol provided a gradual return to activity, starting with slow leash walks for a limited time (5–10 min) in accordance with what could be clinically evaluated (generally until 10 sessions). Then, exercises were introduced, such as beg–stand–beg, lateral step, dancing, and walking backwards. Other types of exercises that could be used in this phase were walking on low obstacles (rail stands), hemi-standing, and walking on slopes (20–40°), both uphill and downhill for about 100 meters [17]. The use of TECAR therapy as a diathermy method is rather innovative in veterinary medicine. TECAR is a diathermic method that acts on deep tissue layers. It is commonly used in human medicine for the treatment of muscle and tendon injuries [26]. Clinical effects result from increases in local blood and lymphatic flow, reduction of pain, and increase in cellular metabolism [27,28]. Ultrasound therapy has also been introduced within the rehabilitation protocol, as previously reported by other authors, for its positive effect on muscle recovery [29,30].

In five patients, the application of counter-current jets was introduced during UWTM to improve muscle tone. Jets were gradually applied to dogs autonomously working in UWTM, at a speed appropriate to the patient’s size and without inducing signs of fatigue, after a minimum training time of 20 min. Jets were introduced gradually for a maximum of 10 min.

Shock wave therapy for chronic lesions has also been reported in the literature as an instrumental therapy for mineralized lesions. For this technique, three applications have been recommended to obtain pain relief [31,32].

If severe iliopsoas injuries, such as wide calcified lesions or recurrent injuries, are diagnosed, surgery can be proposed. Tenotomy of the iliopsoas tendon has been suggested. Three clinical cases have been reported in the literature, in which surgical resolution was the only therapeutic option, with different times of lameness recovery from 8 to 16 weeks after surgery [24,25,33].

Finally, limited data related to the return to sports activity by dogs specifically affected by iliopsoas injury have been reported. Marcellin-Little and collaborators [34] reported that in sports dogs, muscle recovery takes more than 6 weeks. A recent publication has also investigated the time required for muscle healing on the basis of injury grading; the authors reported grade 2 lesions had a very wide range from 3 weeks to 3–6 months [35].

Recently, a human study performed by Pollock and colleagues reported four degrees of muscle trauma classification in 44 athletes [36]. The athletes reported acute injuries, which were diagnosed by magnetic resonance imaging within seven days after trauma and submitted to physiotherapy treatment. The muscles most involved were the long head of the biceps femoris *(Musculus biceps femoris*) and secondary semitendinosus muscle (*Musculus semitendinosus*), the semimembranosus muscle (*Musculus semimembranosus*), and the short head of the biceps femoris. The researchers reported a time to return to sports activities that increased proportionally with the increase in the degree of muscle injury: grades 1 and 2, regardless of location, had an average time of 20 days; grades 3 and 4 had an average time of about 80 days. Although the clinical cases enrolled by Pollock et al. [36] reported muscle injuries involving the thigh region, we have to consider that the iliopsoas muscle in the dog has both postural and active hip movement functions. The iliopsoas injuries observed in the enrolled dogs could be associated with the grade 2 trauma reported by Pollock and collaborators. Those authors reported a mean time of recovery from mild trauma of 20 days [36]; this finding is similar to the mean time in our investigation.

This preliminary study, in our opinion, reports innovative data, although with undeniable limitations. The main limitations are related to the retrospective nature of this investigation, a low number of patients included in the study, the absence of ultrasound investigation in all 10 clinical cases (only five), and the absence of a control group with patients not treated with the physiotherapy protocol.

## 5. Conclusions

Iliopsoas injures are particularly common in sports dogs that practice agility or in disc dogs. Traumatic lesions are generally of a mild-to-moderate grade for which a conservative approach of multimodal physiotherapy could lead to recovery of lameness after at least six sessions of intensive rehabilitation, distributed over a period of 3 weeks. Supported physiotherapy after recovery should always be suggested to the owners to limit injury recurrence and promote muscle strengthening. However, further investigations with a larger sample are warranted to confirm the outcomes reported in this preliminary study.

## Figures and Tables

**Table 1:** Dogs included in the study.

N°	Breed	Age (Years)	Gender	Sports	BCS	Pain P	Pain Ex
1	Border Collie	4	F	Disk Dog	5	Y	N
2	Border Collie	4.5	F	Agility and Sheepdog	5	Y	N
3	Border Collie	5.5	M	Agility	5	Y	Y
4	Mixed breed	6	F	Agility	6	Y	Y
5	Border Collie	12.5	M	None	5	Y	Y
6	Border Collie	4	M	Disk Dog and Obedience	5	Y	N
7	Jack Russell Terrier	7	F	None	5	Y	Y
8	Border Collie	1.5	F	Disk Dog	5	Y	N
9	Belgian Malinois	1	F	None	5	Y	N
10	Border Collie	7	M	Agility dog	5	Y	N

BCS = body condition score; F = female; M= males; Pain P = pain on palpation at first physiatric examination; Pain EX =pain on extension at first physiatric examination; Y = presence of pain; N = absence of pain.

## Data Availability

All data is contained within this article. Interested qualified researchers may request further information by contacting the corresponding author.
